# Patients and Healthcare Providers’ Perspectives on Patient Experience Factors and a Model of Patient-Centered Care Communication: A Systematic Review

**DOI:** 10.3390/healthcare12111090

**Published:** 2024-05-26

**Authors:** Eun-Jeong Kim, Yoo-Ri Koo, Inn-Chul Nam

**Affiliations:** 1 Department of Industry-Academic Cooperation Foundation, The Catholic University of Korea, Seoul 06591, Republic of Korea; dodam.design.research@gmail.com; 2 Department of Service Design, Graduate School of Industrial Arts, Hongik University, Seoul 04066, Republic of Korea; yrkoo@hongik.ac.kr; 3 Department of Otorhinolaryngology-Head and Neck Surgery, Incheon St. Mary’s Hospital, The Catholic University of Korea, Seoul 21431, Republic of Korea

**Keywords:** patient-centered care, patient-healthcare provider perspective, quality of healthcare, health communication

## Abstract

Effective communication between patients and healthcare providers is essential for a positive patient experience (PE), and improving patient-centered care (PCC) involves many factors. This study aimed to (1) identify the factors that affect PE improvement, (2) reflect patients and healthcare providers’ perspectives on the factors’ importance, and (3) present a structural model for improving PCC. A systematic review of empirical studies that specified PE factors was conducted. Studies that did not reflect users’ perspectives and non-empirical studies were excluded. The literature was searched using Google Scholar, PubMed, Web of Science, and the Taylor and Francis online journal. The MMAT 2018 checklist was used to assess bias in the included studies, and frequency, content, and thematic analyses were employed to synthesize the results, yielding 25 articles. The 80 PE factors identified from the analyses were categorized into six categories: Practice, Physical Needs, Psychological Needs, Social Needs, Practical Needs, and Information Needs. From a user perspective, patients emphasized professional, continuous, and comprehensive service delivery, whereas healthcare providers stressed efficient system improvements and positive provider–patient relationships. We propose a structured model for PCC improvement using a service blueprint and system map. The PCC model provides an overview of the interactions and the roles of all stakeholders regarding quality of care to improve healthcare.

## 1. Introduction

### 1.1. Theoretical Background

Patient-centered care (PCC) emphasizes efficient communication between healthcare providers and patients by involving them in the treatment process [1], which is essential for understanding patient experience (PE) and achieving effective care outcomes by reducing physical symptom pain and emotional distress and improving patient satisfaction [2,3,4,5,6]. PE, which refers to a patient’s interaction with the healthcare system [7], enables quality of care through effective communication, respect, dignity, and emotional support [8,9,10,11]. PCC communication is the key to quality care; therefore, there is a need to comprehensively understand the various PE factors that affect PCC. In particular, the concept of PCC has contributed to changing the role of patients from passive service recipients to active participants in treatment-related decision making [12,13]. PCC, defined as “communicative behaviors” that improve the quality of the relationship between healthcare providers and patients based on trust, is closely related to the patient’s informational needs and desire for shared decision making [6,14], playing a vital role in increasing patient understanding, perceiving empathy, and encouraging patient participation [15].

However, McDermott and Pedersen [16] pointed out that although the roles and participation of patients have been highlighted as active collaborators in the provision of medical services, discussions on the various factors affecting service improvement are relatively scarce.

To improve and manage PE qualitatively, examining PE factors within a comprehensive framework is necessary. Moreover, it is vital to understand the relationship between factors and their interaction with or influence on a user’s perspective. The PE factors affecting PCC are markedly diverse and complex. Each stakeholder, including patients, medical staff, and hospital administrators, has different perspectives on these factors and considers them to be of varying importance. Therefore, to provide PCC, it is crucial to adopt a comprehensive view of PE factors from the perspectives of both patients and healthcare providers. Understanding their relative differences and common perspectives can improve medical services in terms of treatment efficiency and user satisfaction by balancing their needs in the treatment process. 

### 1.2. Research on PCC for PE Improvement

Efforts to improve PCC are currently underway, specifically by examining the relationship between various factors from an integrated perspective and developing these results into a model focused on PE throughout the treatment process [17,18]. Specifically, using a systematic model, it is essential to highlight the relationships between PE-related factors and their characteristics. Developing a specific, structured, and systematic model for PCC helps understand the approach and scope of application in PCC [19]. 

The need for integrative and systematic perspectives on PE factors has encouraged the development of various models of PCC over the past 20 years. PCC models have been presented in various forms, including frameworks, typologies, concept maps, and domains (e.g., a concept map of PCC pathways [17], a conceptual map for PCC [19], and a conceptual model of patient empowerment [20]). These models introduce considerations for achieving patient-centeredness through literature and policies [21]. However, many studies only explain models using abstract concepts and are unable to structure said models in a way that facilitates a clear understanding of the relationships, influences, and flows between specific factors. Although difficulties still need to be solved in applying these models to actual medical environments or sites to analyze problems and find solutions, existing models have helped to understand the PE factors related to PCC conceptually.

### 1.3. Study Aim and Research Questions

In order to utilize the systematic review to develop a PCC delivery model, the authors set out to (a) identify PE factors that contribute to the improvement of PCC, (b) identify those PE factors that patients and healthcare providers consider important and are reflected in their perspectives, and (c) construct a model which takes account of the interaction between these PE factors and how they affect the perceptions and role performances of critical stakeholders. 

The specific research questions, derived from the study purpose, are as follows.

RQ1. What are the PE factors that influence the improvement of PCC?

RQ2. What PE factors do patients and healthcare providers consider important, and what are the differences in their perspectives?

RQ3. How can a PCC delivery model be structured based on the roles and interactions of the PE factors and stakeholders?

## 2. Materials and Methods

For this systematic review, we followed the Preferred Reporting Items for Systematic Reviews (PRISMA) guidelines [22] and reported the review results based on the PRISMA 2020 checklist [22]. This review was prospectively registered with the International Prospective Register of Systematic Reviews (PROSPERO) (CRD: 42024499657). 

### 2.1. Eligibility Criteria

The eligibility criteria included peer-reviewed journal articles addressing the PE factors affecting PCC in healthcare services. To understand the PE factors affecting PCC from various stakeholder perspectives, terms such as patient needs, barriers, enablers of PE, evidence, PE improvement, protocol, requirement, and insight were used as search terms. The inclusion criteria were (1) papers published in English and (2) empirical studies with identifiable authors and abstracts. 

Papers whose primary purpose was not to identify PE factors but in which factors were used to evaluate or assess PE or develop prototypes were excluded because the PE factors were not explicitly mentioned. 

### 2.2. Information Sources

The search was conducted between December 2023 and January 2024 using four electronic databases, three scientific databases (i.e., Google Scholar, PubMed, Web of Science), and a publisher’s database provided by Taylor and Francis online. The latter contains a large number of academic papers ranging from social science to design perspectives, thus allowing researchers to flexibly search for information sources from a variety of perspectives. 

### 2.3. Search Strategy

We searched for papers published between 2015 and 2023, spanning the past nine years. The search keywords used were “healthcare”, “service design”, “patient experience”, “patient-centered communication”, “quality improvement”, and “patient-centered care”. Combinations of words (e.g., needs, barriers, evidence, improvement, protocol, requirement, and insight) were included to expand the search scope. Table 1 lists the search terms used in each database. 

### 2.4. Selection Process

Three researchers participated in the data selection to increase inter-rater reliability in researchers’ coding behavior at each decision-making stage in the review process [23]. Two researchers independently screened the titles and abstracts after searching the papers in the four databases and removing duplicates. The results of the first screening were cross-examined to confirm whether the results were the same; if opinions differed, a third reviewer participated in reaching a consensus. In the second full-text review, two researchers independently reviewed the papers and met with a third reviewer to reach a consensus on the selected articles. The researchers manually screened the papers without the use of screening software. The number of studies left for screening after excluding duplicates and items lacking abstracts was low enough to be manageable using manual screening without software. Three researchers spent sufficient time on manual screening to make accurate and precise decisions. 

### 2.5. Data Collection Process

Before data collection, the three researchers who participated in the data selection process set the data collection criteria and items using Microsoft Excel. The two researchers involved in data collection independently collected and organized the data in Microsoft Excel according to the established framework. Subsequently, one reviewer reviewed the discrepancies in the data results, and three researchers worked together to reach a consensus on the final opinion.

### 2.6. Data Items

The characteristics of selected studies included the following: authors, publication year, country, study purpose, target users, study participants, methods used to identify the PE factors, analysis methods, and study results. To present a structured model for PCC, we identified the PE factors mentioned in each paper and sought to determine the perspective from which the identified PE factors were mentioned.

### 2.7. Study Risk of Bias Assessment

To assess the risk of bias, we employed the 2018 version of the MMAT checklist [24], which assesses the quality of mixed-methods empirical studies. The evaluation was divided into qualitative, quantitative, and mixed-methods studies and was conducted independently by two researchers. The evaluation items used included two screening questions for all types of research: five for qualitative studies, five for quantitative descriptive studies, and five for mixed-methods studies. Instead of assigning numerical values, studies were evaluated as low, moderate, or high quality. Two researchers independently evaluated the items for each study based on three responses. After the evaluation was completed, the responses of the two researchers were compared. In the case of disagreement in the evaluation, a third reviewer participated, collected the opinions of the three researchers, and judged the final results. The final assessment result was evaluated as high if all five items received high scores and moderate if 3–4 items received high scores. This review confirmed all studies to be of moderate or higher quality.

### 2.8. Synthesis Methods

Each PE factor extracted from the selected papers was grouped into items with similar meanings, and high-level categories were derived based on the similarities between groups. We derived a three-level hierarchy of categories, subcategories, and PE factors through this process. 

To visualize the patients’ and healthcare providers’ perspectives on the PE factors and the roles and interactions that the providers have with each factor, two service design methods were applied as a novel and effective methodology for structuring and visualizing data. A system map was used to explain PE factor attributes and stakeholder perspectives comprehensively. Specifically, we employed Shostack’s service blueprint to visualize the PCC delivery model, which visually structures intangible interactions between service providers and service users, making it easy to understand and clearly explain the process [25]. 

## 3. Results

### 3.1. Study Selection

**Figure 1:**
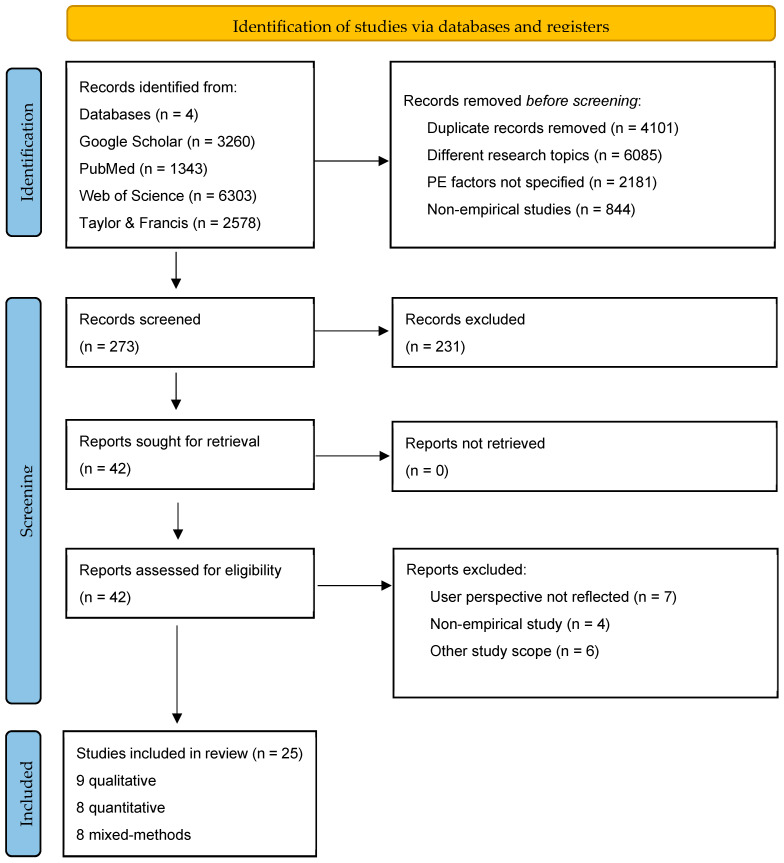
The PRISMA flow diagram of the study.

The initial search yielded 3260 papers from Google Scholar, 1343 from PubMed, 6303 from Web of Science, and 2578 from Taylor and Francis (total = 13,484 papers). After removing duplicates and papers for which no abstract was provided, 4101 records were excluded. Studies not related to improving PE in healthcare services or PE factors were not specifically mentioned, and non-empirical studies were excluded. Of the 273 remaining records, 231 were screened by title and abstract reviews. Subsequently, 42 studies were retrieved and assessed for eligibility. Papers that did not reflect the user’s perspective, were non-empirical, or had a research scope or target too specific to a specific field were also excluded. Finally, 25 studies were included in the review (9 qualitative, 8 quantitative, and 8 mixed-methods studies) (Figure 1).

### 3.2. Study Characteristics

#### General Findings

Most of the 25 studies were conducted in the USA (n = 7), followed by the UK (n = 5) and Australia (n = 4). Three studies were conducted in Canada and several other countries, whereas one study each was conducted in Ghana, Norway, Spain, and Sweden. Regarding target users, studies targeting patients and caregivers were the most common (n = 14). Six studies targeted both patients and healthcare providers; two targeted only healthcare providers; and three included healthcare stakeholders, such as administrators and organization leaders. Participants included patients (n = 10), both patients and healthcare providers (n = 7), multidisciplinary teams (n = 4), healthcare providers (n = 2), and healthcare professionals (n = 2). This shows that in connection with the target group, the participation of the patient group was the highest. 

In terms of study aim, most of the studies (n = 18) identified patients’ needs and perspectives, with four studies related to patients’ engagement and interaction and one study on system improvement. Qualitative studies (n = 9) used methods such as interviews (n = 7), focus group meetings (n = 3), site visits (n = 2), and workshops (n = 1), whereas thematic analysis (n = 7) was the most commonly used approach. Quantitative analyses (n = 8) were conducted to illustrate the statistical results of the survey (100%). In the mixed-methods studies (n = 8), many studies (n = 5) used a combination of interviews and surveys. A free-text comment-based survey (n = 1) and a cohort study (n = 1) were conducted. Mixed-methods studies utilized statistics (n = 4) and content/thematic analysis (n = 5).

Only 8 of the 25 studies presented suggestions for strengthening PCC: suggestion of ideas (n = 3), hierarchy of unmet needs (n = 1), refinement of the existing process (n = 1), screening tool development (n = 1), intervention to be developed (n = 1), and checklist (n = 1). Table 2 presents the overall characteristics of the selected studies, and Table 3 presents the quantified results for each data item.

### 3.3. Risk of Bias in Studies

The 25 selected articles were evaluated for the risk of bias using the MMAT 2018 checklist. The study included nine qualitative, eight quantitative, and eight mixed-methods studies. The evaluation results showed that all selected studies were of moderate or higher quality, with 16 high-quality studies (6 qualitative studies [27,28,29,31,32,33], 4 quantitative studies [35,36,37,38], 6 mixed-methods studies [43,45,46,48,49,50], 9 moderate-quality studies (three qualitative studies [26,30,34], 4 quantitative studies [39,40,41,42], and 2 mixed-methods studies [44,47]). 

### 3.4. PE Factors Affecting PCC

After examining the PE factors related to PCC acquisition in 25 selected studies, 80 factor items were derived. Each item was classified through thematic analysis based on similarities in meaning or content centered on keywords. The 80 PE factors were classified into 20 subcategories (system, tool, coordination, care plan, management, skill, physical support, physical symptoms, emotional support, psychological symptoms, communication/interaction, support and involvement, respect, access to information, access to care, access to services, environment, knowledge, information content and extent, and education). The 20 subcategories were re-clustered with similar topics, and six higher-level categories were presented: *Practice* (n = 22), *Physical Needs* (n = 3), *Psychological Needs* (n = 5), *Social Needs* (n = 14), *Practical Needs* (n = 17), and *Information Needs* (n = 19) (Table 4). 

### 3.5. Patients and Healthcare Providers’ Perspectives on PE Factors

The perspectives of patients and healthcare providers, active co-actors in PCC delivery and service improvement, were compared to determine the most critical PE factors (Table 5). 

In *Practice*, continuity was mentioned in nine papers as an essential factor by both patients and healthcare providers. Following this, four papers mentioned treatment service at home, and three mentioned healthcare providers’ workload, plan development, patient involvement, and discharge/alteration level change as significant factors affecting PCC. Patients mentioned comprehensiveness, professional skill, and diagnosis as essential factors. In contrast, healthcare providers emphasized factors related to the administrative system, such as complex and fractured systems, lack of providers’ voices, and medicine management. In other words, the critical factors identified were clear language, explanations, advice, techniques that patients could easily understand and use, and medical staff treatment ability and management, all of which directly impact a patient’s treatment. 

In the *Physical Needs* category, both patients and healthcare providers viewed minimizing pain or discomfort, emergency care, and physical symptoms as essential for PCC, revealing that meeting *Physical Needs* is crucial.

Regarding *Psychological Needs*, both patients and healthcare providers identified assistance and psychological symptoms related to patients’ emotions and relationships as the most critical factors. In addition, patients mentioned the emotional impact of their psychology on their daily lives as a critical factor. This illustrates that the psychological impact of the treatment journey on the patient is substantial and that it is necessary to focus not only on managing the physical aspects of the patient’s treatment but also on the psychological aspects. 

Among the various PE factors included in *Social Needs*, both patients and healthcare providers mentioned forming trusting relationships most frequently. Commonly mentioned factors included support and involvement, assistance for daily living, having friends or family, discussing patient concerns with clinicians, finance, and insurance support. Patients considered providing information to professionals and service providers’ interpersonal skills influential. At the same time, medical staff emphasized the need to avoid poor practices (e.g., indifference to patients’ personal situations, age differences, and disagreements) and the lack of shared information/empathy. This implies that patients value two-way interactive communication between doctors and patients during the treatment process. Additionally, the results revealed that healthcare providers attach importance to being empathetic and understanding the patient, addressing the communication gap resulting from the age difference between healthcare providers and patients, as well as the need to coordinate opinions on the direction of treatment between the family (caregiver) and the patient. Finally, while patients focus on communication with medical staff while providing and receiving medical services, healthcare providers believe that additional factors, such as age and family opinions, can affect the medical service experience. 

About *Practical Needs*, both patients and healthcare providers frequently cited information access as an essential factor in PCC. Subsequently, waiting, availability, catering, schedule, parking, transportation, contact, and waiting rooms were identified as the significant factors. Patients highlighted timing, conference rooms, noise, and hygiene, whereas medical staff highlighted staff capacity, limited space, signposts, and unfamiliar environments as essential factors. Patients preferred an environment in which they could escape the noise, secure their privacy, and focus on discussing and managing care, while healthcare providers prioritized creating an environment that provides efficient medical services and psychological stability for patients in an unfamiliar hospital environment. 

Regarding *Information Needs*, both patients and healthcare providers mentioned the importance of information content. Training and education, patient knowledge, access to more and up-to-date information, and information usefulness were identified as essential factors in PCC improvement. Patients considered misconceptions, self-perception, awareness, information consistency, information usefulness, and information data relatively important, whereas healthcare providers emphasized clinicians’ communication knowledge and building resilience. In other words, while patients focused on the qualitative aspects of information, such as clearly understanding the care information received from medical staff and receiving consistent and valuable information, healthcare providers placed significant emphasis on the method and system used to deliver information to patients, stressing the ability to convey information to patients in a comprehensive manner and the importance of education in ensuring consistent long-term patient treatment and management.

In summary, patients and healthcare providers viewed the following as common factors for obtaining PCC: provision of continuous medical services, home service after discharge, plan development, and patient involvement in the *Practice* category; alleviation of patient pain and preparedness for emergencies in the *Physical Needs* category; and emotional assistance and the strengthening of relationships with patients in the *Psychological Needs* category. Regarding *Social Needs*, the following were common factors in obtaining PCC: forming trusting relationships between patients and doctors, assistance for daily living and discussion concerns, having friends or family, and financing and insurance. *Practical Needs* emphasized access to treatment information and facilities, whereas *Information Needs* revealed that controlling the quantity and quality of information to improve patient understanding and education were key factors.

Looking at the differences between these two groups, in contrast to patients who highlighted the functional aspects directly related to treatment (provision of consistent and comprehensive services, emotional management, noise and hygiene control, and provision of their information to providers), healthcare providers focused on the relative importance of addressing structural problems (complex and fractured systems, staff capacity, limited space, and wayfinding) of the hospital system and the healthcare environment.

In addition, patients chose respect for their feelings during the treatment process and appropriate responses as the main factors (the impact of one’s negative psychology and management and the attitude of medical staff toward patients). Conversely, healthcare providers considered essential factors related to forming positive relationships with patients such as building empathy with patients and the communication skills that lead to such relationships.

### 3.6. A Structured and Systematic Model for Explaining the Interaction and Workflow of PCC Delivery

**Figure 2:**
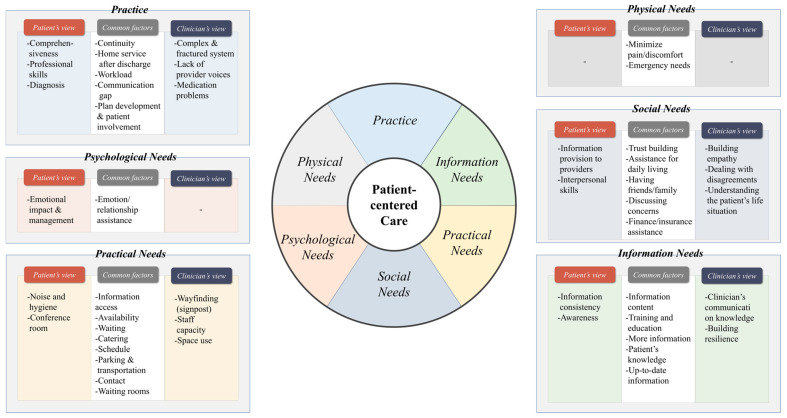
Six categories of PE factors affecting PCC and perspectives of patients and healthcare providers.

Figure 2 illustrates patients’ and healthcare providers’ perspectives regarding the identified PE factors. PCC is influenced by various factors according to six categories. For each category, in addition to the items commonly considered necessary by patients and healthcare providers, the factors prioritized by each user differed slightly.

**Figure 3:**
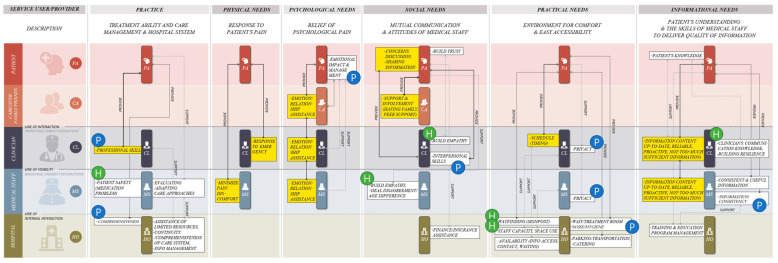
Service blueprint: A structured system model for PCC delivery.

The provider of each factor and the service delivery flow were defined to structure the PCC delivery model based on Figure 2. This resulted in a service blueprint (structural system model), as shown in Figure 3. The blueprint introduced by Shostack [30] represents the interaction between service providers and users according to the journey stages. In this study, Shostack’s model was modified to suit our results. For each of the six PE categories, the roles of patients (service users) and healthcare providers (service providers) were diagrammed, as were the interactions between users. The vertical axis represents the stakeholders involved in PCC delivery and is divided into five user types: patients, caregivers, doctors, medical staff, and hospitals. The horizontal axis is divided into six PE categories. The PE factor for each category is linked to the service provider, and its location on the map is allotted considering the interaction between the users. The direction of service provision flow between each factor is indicated by arrows. The yellow box represents the PE factor provided to the patient directly, and the factors considered necessary by the patient and healthcare provider are marked separately as “P” in a blue circle and “H” in a green circle.

For example, in *Practice*, “professional skill” is a PE factor directly provided to the patient by a clinician. For “patient safety regarding medication” and “evaluating care approaches”, medical staff are the primary providers of services. In the former case, patients and healthcare providers consider “professional skills” and “patient safety” paramount, respectively. “Evaluating care approach” is a factor that both patients and healthcare providers deem essential. “Comprehensiveness” is a factor a hospital must provide and represent, and patients consider it particularly important.

**Figure 4:**
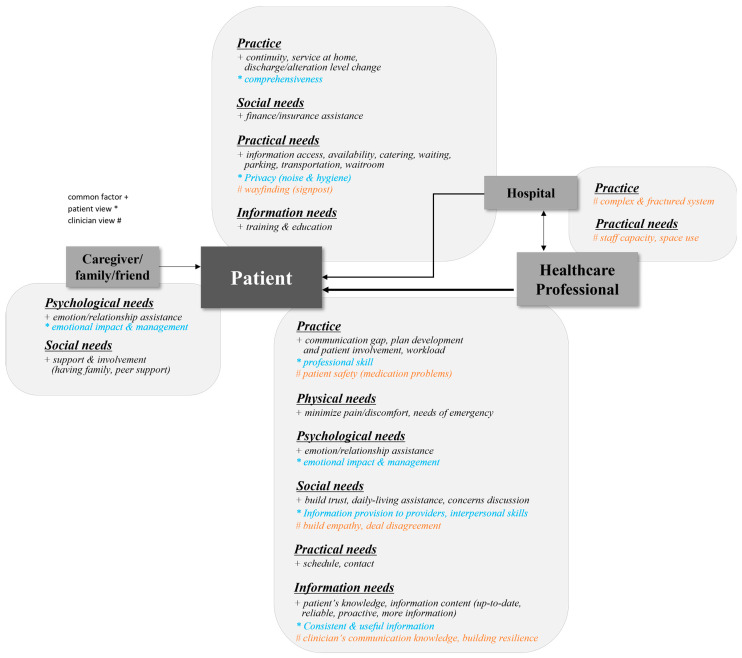
A system map of PCC delivery showing the influence and relationship between patients and service providers focusing on PE factors.

While Figure 3 expresses the interaction of PE factors affecting PCC by PE category, Figure 4 presents the factors that caregivers, healthcare providers (clinicians and medical staff), and hospitals are responsible for in PCC. For example, the improvement of complex and fractured systems (*Practice*) and staff capacity and space use (*Practical Needs*) shown on the right side of the figure correspond to factors that are closely related to both hospitals and healthcare providers. These factors have an indirect rather than a direct effect on patients. This model shows that improving these factors for effective PCC requires cooperation between hospitals and healthcare providers.

## 4. Discussion

### 4.1. Main Findings

This review found that most selected studies focused on targeting patients and understanding their needs and perspectives to improve PCC. Identifying patients’ unmet needs and reflecting them in healthcare services is essential for effective PCC delivery. However, diagnostic and treatment processes involve two-way communication between service users and healthcare providers. Therefore, looking at healthcare service delivery only from the patient’s perspective provides an incomplete picture of how to improve PCC. It is essential to also consider the perspectives of healthcare providers who interact with patients.

Although some of the selected studies demonstrated attempts to listen to the opinions of both patients and healthcare providers, there were many cases where only patients participated in the research, and most studies focused on understanding solely patients’ needs and perspectives. Conversely, only one study aimed to improve the healthcare system.

In addition to understanding the factors that affect PCC improvement, we also examined the specific relationships between the PE factors in terms of which these factors were related to the PCC process, who took the lead in shaping the process, and what interactions occurred between each factor to better understand the PCC system. Identifying these structural relationships can provide an overview for the understanding and improvement of complex healthcare services [26].

More than half of the reviewed papers did not present tangible solutions regarding paths to improve PCC. Thus, although many studies make valuable contributions by identifying patient needs and barriers to change, they often fail to suggest ways to overcome them. As McDermott and Pedersen [16] pointed out regarding the lack of discussion on factors influencing service improvement for effective PCC delivery, it would be helpful to have a systematic model of who should lead in improving these factors and how best to approach them.

### 4.2. Importance of Understanding PE Factors and Reflecting Service Providers’ Perspectives

Although the reviewed studies addressed patient needs and perspectives on PCC to some degree, they did not adequately reflect the diversity of PE. This study examined multiple factors affecting PCC from the selected studies by classifying them into practice, physical, psychological, social, practical, and informational aspects. Based on these categories, we sought to develop an integrated perspective that takes account of both service provider and service user perspectives.

By widening the focus in this manner, we aimed to understand better the specific roles and interactions of users involved in PCC delivery. Our model shows that the key factors, according to service users, were the functional aspects of the service (professionalism, continuity, and comprehensiveness), including efficient and polite treatment by medical staff that is consistent and comprehensive, respects patients’ psychological needs, and protects their privacy. Conversely, healthcare providers saw the critical factors as the service’s structural aspect (efficiency) and the quality of the relationship with the patient, leading to a dual concern with the hospital system and environment and the capacity to form positive relationships with patients. This difference in the two groups’ perspectives may not be apparent on either side, and a better understanding would lead to better doctor–patient relationships, improved communication of treatment information, and greater patient participation in decisions and treatment compliance, as suggested by Hong and Oh [15].

### 4.3. Systematic Model for PCC Delivery

This study presented the PCC model in two formats: PE factors and stakeholder perspectives. The former focuses on the role and interaction of the factors in each of the six categories and how the relevant factors provide the service (Figure 3). The stakeholder-centered system model (Figure 4) adopts a user perspective on the factors each service provider focuses on and with whom they interact to provide each factor. In short, by presenting the PCC model from the perspective of PE factors and stakeholders, we attempted to increase its utilization in healthcare settings.

The authors proposed a PCC model in the meaningful form of a service blueprint and system map, which tangibly visualized a complex system centered on service providers’ roles and interactions with service users. While most of the selected studies did not present recommendations for improving PCC, the model developed in this review provides a visual understanding of the structure and flow of the hospital system and can serve as a practical guide for service improvement.

### 4.4. Strengths and Limitations

This study has certain limitations. First, we reviewed only four databases and papers published over a limited period. Thus, some studies that met the criteria of our review may have been omitted. Second, although the PCC model presented in this study was developed after comprehensive review and analysis, its content must be verified by actual users in the field. Field testing is also required to evaluate the use of the model and suggest practical PCC improvements. The PCC model can be refined and further developed through ongoing empirical studies of these matters to maximize its usefulness. Third, the care delivery models, healthcare settings, financial burden of patients, or patient–physician relationships identified in the review may be influenced by different factors depending on the country or setting. However, we could not control for possible study differences, which may cause comparability issues during the PCC model-building process. Despite the above limitations, the results of this study have a particular strength in that they present an integrated and practical framework that comprehensively reflects aspects of PE factors and visualizes the PCC delivery system based on the perspectives of both patients and healthcare providers.

## 5. Conclusions

For promoting PCC, it is necessary to understand the interactions among various PE factors. In this process, multiple stakeholders, including patients, caregivers, doctors, and other hospital staff, shape and are affected by the different factors identified in our PCC model. For medical service delivery improvement, it is essential to understand the interrelationships and the flow of action affected by these complex factors. This study presents a novel model of patient-centered healthcare services by analyzing the factors that directly and indirectly affect PCC and identifying and adjusting the role, interactions, and influence of healthcare service users as coproducers of care. The PE factors model in the current study provides critical insights to increase the understanding of researchers and hospital officials interested in promoting PCC. Our paper makes a unique contribution: it goes beyond simply analyzing and synthesizing the results of the systematic review and incorporates key elements into a visual structured PCC model. Additionally, we hope it will serve as a helpful tool leading to deep understanding and active discussion among stakeholders in analyzing problems and seeking solutions to improve PCC services in actual medical settings.

## Figures and Tables

**Table 1:** Search strategy for systematic literature review.

Electronic Databases	Search Terms
Google Scholar (n = 3260)	“healthcare service design” AND “co-design” OR “experience-based co-design” AND “quality healthcare” OR “quality improvement” AND “patient experience” OR “patient perspectives” OR “patient-centered care”
PubMed (n = 1343)	“healthcare, service design” AND “patient experience” OR “patient centered” OR “co-design” AND “quality improvement”
Web of Science (n = 6303)	ALL = (“healthcare, service design” AND “patient experience” OR “patient centered” OR “co-design” AND “quality improvement”)
Taylor and Francis Journal (n = 2578)	[All: “healthcare, service design”] AND [All: “patient experience”] OR [All: “patient centered”] OR [All: “co-design”] AND [All: “quality improvement”]
Total	N = 13,484

**Table 2:** Characteristics of selected studies.

No.	Authors (Year)	Country	Study Purpose	Target User (Perspective)	Participants	Methods	Analysis	Solutions	MMAT
Qualitative Study (n = 9)									
1	Powell et al. (2016) [26]	USA	Analysis of patient barriers to healthcare engagement and suggestions for improvement	Patients (general)	Interprofessional team members	Focus groups and semi-structured interviews	Content analysis	Suggestion of ideas	Moderate
2	Coy et al. (2019) [27]	UK	Insight identification to meet patient/families’ emotional needs	Children and families suffering from burn injury	Families, practitioners	Interviews, short films, focus group events	Video-based analysis, thematic analysis	Film and suggestion of ideas	High
3	Litchfield et al. (2017) [28]	UK	Development and implementation of strategies for patient satisfaction and efficiency of existing process	Patients and healthcare providers	Patients, medical staff	Focus groups	Thematic -framework analysis	Refinement of existing process	High
4	Kværner et al. (2020) [29]	Norway	Identification of unmet needs for person-centered care and insights for improvement	Patients and medical staff	Multidisciplinary stakeholders (patients and next of kin, health partner employees, decision-makers, representatives from health partners, user organizations, innovation advisors)	Workshops	Stakeholder analysis	Three categories of unmet needs (for scenario building)	High
5	Agha et al. (2018) [30]	USA	Identification of patient engagement barriers and solutions	Medical staff	Medical staff	Interviews, site visits	Thematic analysis	x	Moderate
6	Scott et al. (2017) [31]	USA	Identification of facilitators and barriers to implementation of transitional care services in health systems	Leaders of healthcare organizations, patients, families	Multidisciplinary team (the leadership team, transitional care team, internal stakeholders, community partners, patients and family caregivers)	Interviews, site visits, observations	Thematic analysis	x	High
7	Clarke et al. (2018) [32]	USA	Identification of information needs of primary care patients for the improvement of clinic visit notes (tool)	Primary care physician, patients	Outpatients	Interviews	Thematic analysis	x	High
8	Cardenas et al. (2021) [33]	USA	Identification of patients’ and caregivers’ barriers to home-based palliative care and their recommendations for improvement	Patients, caregivers eligible for palliative care	Patients, proxies, caregivers	Interviews	Thematic analysis	x	High
9	Twamley et al. (2023) [34]	UK	Prioritization areas for improvement in care and services for patients	Patients	Patients, family/friends, hospital staff	Experience-based codesign (EBCD), observation, interviews, film development, feedback/joint event	Thematic analysis	x	Moderate
Quantitative Study (n = 8)									
10	Fitch et al. (2021) [35]	Canada	Review of main challenges of adolescent and young adults (AYA) cancer survivors and their suggestions for care improvement	AYA cancer survivors (18–34 years) and patients	AYA cancer survivors (18–34 years) and patients	Survey	Content analysis	Suggestion of ideas	High
11	Schäfer et al. (2015) [36]	31 European countries, Australia, Canada, and New Zealand	Investigation of patients’ perceptions of improvement potential in primary care	Patients	Patients of general practitioner	Survey	Statistics, evaluation questionnaire	x	High
12	Fradgley et al. (2016) (a) [37]	Australia	Identification of patients’ and health professionals’ quality improvement preferences	Patients and healthcare professionals	Outpatients, healthcare professionals	Survey	Statistics	x	High
13	Fradgley et al. (2016) (b) [38]	Australia	Identification of patients’ preferences of initiatives for change	Outpatients	Outpatients	Survey	Statistics	x	High
14	Hoven et al. (2018) [39]	Sweden	Identification of information needs and satisfaction with provided information	Survivors and families after childhood central nervous system (CNS) tumor treatment	Childhood CNS tumor survivors, parents	Self-report questionnaire	Statistics	x	Moderate
15	Hwang and Warshaw (2019) [40]	USA	Understanding the values and needs of the patients	Geriatric practitioners	American Geriatrics Society (AGS) members	Descriptive survey	Statistics	x	Moderate
16	Amoah et al. (2018) [41]	Ghana	Identification of the barriers to therapeutic communication among nurses and patients	Nurses, patients	Nurses and patients at local public hospital	Survey	Statistics	x	Moderate
17	Hall et al. (2021) [42]	Australia	Identification of the potential intervention to be improved for patient-centered care	Healthcare leaders, researchers, patients	Cancer patients with oncology treatment	Survey	Statistics	x	Moderate
Mixed-Methods Study (n = 8)									
18	Fitch et al. (2019) [43]	Canada	Identification of unmet needs and experiences of cancer survivors for survivorship program improvement	Healthcare providers, patients	Cancer survivors between 1 and 3 years post-treatment	Literature review, interviews, survey	Statistics	x	High
19	Fradgley et al. (2014) [44]	Australia	Development and testing of a web-based survey to identify and prioritize patient-centered initiatives in chronic disease outpatient services	Patients	Oncology patients	Literature review, feedback from expert, pilot study (survey) to test feasibility, survey	Statistics	x	Moderate
20	Agard et al. (2018) [45]	Denmark, Netherlands	Analysis of family perspectives for identifying improvement opportunities in the intensive care unit (ICU)	Family members of patients with ICU stay	Family members of patients with ICU stay	Survey (free text comments)	Content analysis	x	High
21	Martinez-Guiu et al. (2021) [46]	Spain	Identification of patients’ attitudes, experiences, and needs	Chronic obstructive pulmonary disease (COPD) patients	Patients, healthcare professionals	Interviews, survey	Statistics, inductive analysis	x	High
22	Creutzfeldt et al. (2015) [47]	USA	Identification of palliative care needs for patients and their families	Patients in neuro ICU and families	Clinical team in the neuro ICU	Literature review, expert discussion, cohort study	Thematic analysis	Screening tool as a checklist	Moderate
23	Bowie et al. (2015) [48]	UK	Review of existing criteria for selecting “always events” (AEs) and generating a candidate list of AE examples to introduce the concept of AE application	Patients	Patients of primary care settings from 13 practices	Interviews and questionnaire	Content analysis, statistics	x	High
24	Stevens et al. (2018) [49]	UK	Identification of unmet needs among AYA cancer patients for service development	Local healthcare leaders/administrators	AYA patients, families, networkers, professionals	Survey, interviews, focus groups	Requirement management method (engineering and software development system)	Intervention development areas (software)	High
25	Rose et al. (2022) [50]	Canada	Consensus based development of a quality improvement checklist for patients	Patients/family	ICU interprofessional team, patients, family members	EBCD, systematic review, semi-structured video/phone interviews, touchpoint video production, modified delphi, consensus meeting	Content analysis	A quality improvement checklist	High

**Table 3:** Subject scope of the selected studies for PCC.

Category	Results (Total n = 25)	
Country	USA (n = 7, 28%), UK (n = 5, 20%), Australia (n = 4, 16%), Canada (n = 3, 12%), multiple countries (n = 2, 8%), Ghana (n = 1, 4%), Norway (n = 1, 4%), Spain (n = 1, 4%), Sweden (n = 1, 4%)	
Target user	Patients/caregivers (n = 14, 56%), patients and healthcare providers (n = 6, 24%), healthcare administrators/organization leaders (n = 3, 12%), healthcare providers (n = 2, 8%)	
Participants	Patients (n = 10, 40%), patients and healthcare providers (n = 7, 28%), multidisciplinary teams (n = 4, 16%), healthcare providers (n = 2, 8%), healthcare professionals (n = 2, 8%)	
Study scope	Patients’ needs and perspectives (n = 19, 76%), patients’ engagement and interaction (n = 4, 16%), system improvement (n = 1, 4%), checklist development (n = 1, 4%)	
Methods and analysis (multiple methods were counted individually)	Qualitative	Interview (n = 7), focus group meeting (n = 3), site visit (n = 2), workshop (n = 1), short film (n = 2), observation (n = 2), feedback/joint event (n = 1)
	Quantitative	Survey (n = 8)
	Mixed-methods	Literature review/interview/survey (n = 2), survey with free text comments (n = 1), interview/survey (n = 2), literature review/expert discussion/cohort study (n = 1), survey/interview/focus group meeting (n = 1), literature review/interview/video production/delphi/consensus meeting (n = 1)
Solution	None (n = 17, 68%), suggestions of ideas (n = 3, 12%), hierarchy of needs/intervention area (n = 2, 8%), refinement to existing process (n = 1, 4%), screening tool development (n = 1, 4%), checklist (n = 1, 4%)	

**Table 4:** PE factors affecting PCC.

Category	Subcategory	PE Factors
*Practice* (n = 22)	System (n = 6)	Complex and fractured system (lack of uniformity, out-of-date, slow system) [26,30]
		Lack of expertise [29]
		Underutilized care program/system [31]
		Limited resources [31]
		Workload, overwork, staffing (overburden) [30,34,41]
		Bureaucratic culture (demands of national priority, lack of providers’ voices) [30]
	Tool (n = 2)	Alert system/tool [28]
		Simplified tools and services [46,50]
	Coordination (n = 4)	Continuity [34,36,38,42,43,44,45,48,49]
		Communication gap [26,31,37]
		Uniform implementation [31]
		Comprehensiveness [36]
	Care plan (n = 6)	Service at home [26,27,31,34]
		Prioritizing care service [31]
		Medicine management [26]
		Plan development and patient involvement [32,34,50]
		Care management [34,48]
		Discharge/alteration level change [26,34,45]
	Management (n = 2)	Information management [31,45]
		Evaluating and adapting care approaches [31,50]
	Skill (n = 2)	Professional skills (care and treatment) [45]
		Diagnosis [46]
*Physical Needs* (n = 3)	Physical support (n = 2)	Minimize pain or discomfort, physical symptoms help [38,44,50]
		Needs of emergency [34,46]
	Physical symptoms (n = 1)	Physical symptoms; pain, physical discomfort [41,47]
*Psychological Needs* (n = 5)	Emotional support (n = 2)	Assistance (emotion/relationship); emotional support [31,37,38,44,48,49,50]
		Emotional management [46]
	Psychological symptoms (n = 3)	Isolation; fear; anxiety; psychological symptoms; too overwhelmed [27,34,40,41,46,47]
		Feeling of patient insecurity [29]
		Emotional impact [27]
*Social Needs* (n = 14)	Communication/interaction (n = 7)	Providers not understanding patients’ life situations [26]
		Patient concerns discussion with clinician; all your concerns are addressed [37,38,44]
		Form trusting relationships; interactions and relationships [26,31,34,36,38,43,44,48]
		Talk about worries for the future [49]
		Age difference [41]
		Disagreement [47]
		Providing information to professionals [45]
	Support and involvement (n = 5)	Assistance (daily living) [34,37,38,44]
		Support and involvement [31,36,38,42,44]
		Having friends or family; families to be present at the bedside [34,38,44,45]
		Practical support, finance, and insurance [26,34,43]
		Social/peer support [47,49]
	Respect (n = 2)	Interpersonal skills [45]
		Share information, lack of empathy/interest/respect [30,41]
*Practical Needs* (n = 17)	Access to info (n = 1)	Information access [27,28,34,37,42,43,45]
	Access to care (n = 8)	Timing [33]
		Waiting [28,37,38,42,44]
		Schedule [28,37,38,44]
		Parking [37,38,42,44]
		Transportation (access) [37,38,40,44]
		Contact [37,38,44]
		Funding/staff capacity [40]
		Availability [30,36,43,48,50]
	Access to service (n = 1)	Catering [37,38,42,44,45]
	Environment (n = 7)	Waiting rooms [38,44,45]
		Treatment rooms [38,44]
		Conference rooms [45]
		Limited space (teams were not seated with each other, multiple teams were sharing rooms) [30]
		Signposting [49]
		Unfamiliar environment [41]
		Noise and hygiene [45]
*Information Needs* (n = 19)	Knowledge (n = 6)	Knowledge/understanding; patients’ skills or knowledge [33,34,38,40,44]
		Misconception [33]
		Self-perception [33]
		Invisible problems (cognitive) [29]
		Awareness [46]
		Clinicians’ communication knowledge [41]
	Information (content and extent) (n = 10)	Up-to-date information [37,38,42,44]
		Information content [37,38,42,44,45,49,50]
		Information consistency [27]
		More information [34,38,39,44,49]
		Alerting cognitive effects of treatment (care info) [49]
		Information extent, insufficient information/too much information without proper explanation [26,39]
		Information usefulness [34,39,49]
		Information data [32]
		Inadequate review (content) [31]
		Proactive information [26]
	Education (n = 3)	Training and education; education [30,31,43,46,49,50]
		Self-management skills/education [49,50]
		Building resilience [49]

**Table 5:** Comparative summary of the perspectives of the patients and healthcare providers.

Category	Common Factors	Patient’s Perspective	Healthcare Provider’s Perspective
*Practice*	Lack of expertise [29], Underutilized care program/system [31], Limited resources [31], Workload [30,34,41], Alert system/tool [28], Simplified tools and services [46,50], Continuity [34,36,38,42,43,44,45,48,49], Communication gap [26,31,37], Uniform implementation [31], Information management [31,45], Evaluating and adapting care approaches [31,50], Service at home [26,27,31,34], Prioritizing care services [31], Plan development and patient involvement [32,34,50], Care management [34,48], Discharge/alteration level change [26,34,45]	Comprehensiveness [36], Professional skill [45], Diagnosis [46]	Complex and fractured system [26,30], Bureaucratic culture (lack of providers’ voices) [30], Medicine management [26]
*Physical Needs*	Minimize pain or discomfort [38,44,50], Emergency needs [34,46], Physical symptoms [41,47]	-	-
*Psychological Needs*	Assistance (emotion/relationship) [31,37,38,44,48,49,50], Psychological symptoms [27,34,40,41,46,47], Feeling of patient insecurity [29]	Emotional management [46], Emotional impact [27]	-
*Social Needs*	Patient concerns about discussions with clinicians [37,38,44], Form trusting relationships [26,31,34,36,38,43,44,48], Talk about future worries [49], Assistance (daily living) [34,37,38,44], Support and involvement [31,36,38,42,44], Having friends or family [34,38,44,45], Practical support (finance and insurance) [26,34,43], Social/peer support [47,49]	Providing information to professionals [45], Interpersonal skills [45]	Providers do not understand patients’ life situations [26], Dealing with disagreement [47] Age difference [41], Share information/lack of empathy [30,41]
*Practical* *N* *eeds*	Information access [27,28,34,37,42,43,45], Timing [33], Waiting [28,37,38,42,44], Schedule [28,37,38,44], Parking [37,38,42,44], Transportation [37,38,40,44], Contact [37,38,44], Availability [30,36,43,48,50], Catering [37,38,42,44,45], Waiting rooms [38,44,45], Treatment room [38,44]	Conference room [45], Noise and hygiene [45]	Funding/staff capacity [40], Limited space [30], Signposting [49], Unfamiliar environment [41]
*Information needs*	Patient’s knowledge [33,34,38,40,44], Misconception [33], Self-perception [33], Cognitive problems [29], Up-to-date information [37,38,42,44], Information content [37,38,42,44,45,49,50], More information [34,38,39,44,49], Alerting cognitive effects of treatment [49], Information extent/insufficient information [26,39], Information usefulness [34,39,49], Inadequate review [31], Proactive information [28], Training and education [30,31,43,46,49,50], Self-management skills/education [49,50]	Awareness [46], Information consistency [27], Information data [32]	Clinician’s communication knowledge [41], Building resilience [49]

## Data Availability

The data that support the findings of this study are available on request from the corresponding author. The data are not publicly available due to privacy or ethical restrictions.
